# Assessing trends and burden of occupational exposure to asbestos in the United States: a comprehensive analysis from 1990 to 2019

**DOI:** 10.1186/s12889-024-18919-7

**Published:** 2024-05-27

**Authors:** Xujun Li, Xin Su, Li Wei, Junhang Zhang, Donglei Shi, Zhaojun Wang

**Affiliations:** 1 Department of Thoracic Surgery, The Seventh Affiliated Hospital, Sun Yat-sen University, Shenzhen, China; 2 Department of Respiratory, Hainan Hospital of PLA General Hospital, Sanya, China

**Keywords:** Asbestos, Annual percentage changes, Annual average percentage changes, Death, Age-standardized rates

## Abstract

**Background:**

This study aimed to analyze the trends and burden of occupational exposure to asbestos in the United States (U.S.) from 1990 to 2019, focusing on mortality rates, geographic distribution, age and sex patterns, and causes of death.

**Methods:**

Data on the number of deaths attributable to occupational exposure to asbestos were collected from 1990 to 2019 in the U.S. Joinpoint analysis was conducted to assess trends over time, and regression models were applied to calculate annual percentage changes (APC) and annual average percentage changes (AAPC). Geographic distribution was examined using mapping techniques. Age and sex patterns were analyzed, and causes of death were identified based on available data.

**Results:**

From 1990 to 2019, the overall number of deaths due to occupational exposure to asbestos in the U.S. increased by 20.2%. However, age-standardized mortality rates (ASMR) and age-standardized disability-adjusted life years (DALYs) rates (ASDR) exhibited a decline over the same period. Geographic analysis revealed differences in the number of deaths across states in 2019, with California reporting the highest number of fatalities. Age-specific mortality and DALYs showed an increase with age, peaking in older age groups. Tracheal, bronchus, and lung cancer were the leading causes of death attributed to asbestos exposure, with increasing trends observed over the past five years.

**Conclusion:**

The study highlights significant trends and burden in occupational exposure to asbestos in the U.S., including overall increases in mortality rates, declining ASMR and ASDR, geographic disparities, age and sex patterns, and shifts in causes of death. These findings underscore the importance of continued monitoring and preventive measures to mitigate the burden of asbestos-related diseases.

**Supplementary Information:**

The online version contains supplementary material available at 10.1186/s12889-024-18919-7.

## Background

Asbestos, a group of naturally occurring minerals renowned for their heat resistance and durability, has been extensively utilized in various industries worldwide [1]. Despite its industrial utility, asbestos is now recognized as a potent carcinogen, posing significant health risks to individuals exposed to its fibers. In 1977, the International Agency for Research on Cancer determined that asbestos meets the criteria for being a human carcinogen [2, 3]. All forms of asbestos have carcinogenic effects on humans, including lung and laryngeal cancer, among others. Additionally, it can cause non-malignant conditions such as asbestosis, pleura thickening, and pleura plaques [4]. An estimated 125 million people globally are thought to be exposed to asbestos at work at this time [4, 5]. Asbestos is responsible for approximately 255,000 deaths per year (with a range of 243,223 to 260,029), with 233,000 deaths resulting from work-related exposure (ranging from 222,322 to 242,802) [6]. Mesothelioma is a malignant tumor that can be attributed to asbestos exposure in at least 80% of cases [7], according to the Global Burden of Disease (GBD), approximately 29,300 people worldwide died from mesothelioma in 2019 [8, 9], doubling since 1990 [10]. Due to the long incubation period of asbestos-related diseases, even if the use of asbestos were to cease now, it would take several decades for the number of related deaths to decrease [5, 11]. According to an article published by Soeberg et al. in 2018, although Australia had implemented a comprehensive asbestos ban in 2003, it was not until 2018, almost fifteen years later, that Australia had just seen a peak in the prevalence of asbestos-related diseases, and the risk of asbestos exposure continues to exist [12].

South Korea acknowledged the hazards of asbestos in 1993, but it wasn’t until 16 years later, in 2009, that a complete ban was enacted [13]. In the UK, while asbestos policy was established in 1932, a full ban was not implemented until 1999 [14]. New Zealand took 13 years to adopt a policy on asbestos that paralleled Australia’s approach [14, 15]. Despite approximately 60 countries having completely banned the use of asbestos and the World Health Organization’s continuous calls for countries to cease its usage [16], the implementation of new bans has recently slowed down [9]. Asbestos remains widely utilized today [6]. However, due to health and liability concerns associated with asbestos use, its utilization in the United States (U.S.) is gradually decreasing. In 2023, it’s estimated that the U.S. consumed approximately 150 tons of chrysotile asbestos [17]. According to GBD 2016, the estimated number of deaths from pleura mesothelioma caused by asbestos in the U.S. is 3,282 [6]. Currently, there exists a lack of detailed estimates regarding the number of deaths caused by asbestos-related diseases in the U.S., as well as the distribution of deaths attributed to different causes.

In this study, we aim to analyze asbestos-related disease trends and burden in the U.S. over the period from 1990 to 2019. Our focus is on identifying temporal patterns in mortality rates, geographic distribution of disease burden, age and sex-related disease patterns, and changing causes of death. This will deepen our understanding of asbestos-related diseases’ landscape and support the development of prevention and intervention strategies. We hope that the U.S. will recognize the hazards of asbestos use, implement a complete ban on its usage.

## Materials and methods

### Data sources

The GBD study stands as an ongoing global collaborative epidemiological endeavor, meticulously assessing the burden of diseases, injuries, and risk factors across 204 countries and regions by gender and age group. Currently, it incorporates data spanning from 1990 to 2019. The GBD study provides assessments of adjusted life years for mortality and disability attributed to 369 diseases and injuries, alongside 87 risk factors [18, 19]. For this research, data concerning the number of deaths, disability-adjusted life years (DALYs), age-standardized DALYs rates (ASDR), and age-standardized mortality rates (ASMR) associated with all causes of occupational exposure to asbestos in the U.S. from 1990 to 2019 were procured from the GBD 2019 dataset (http://ghdx.healthdata.org/gbd-results), regarding particular data extraction techniques, refer to additional file 1. Simultaneously, we analyzed the unique burden of diseases resulting from occupational exposure to asbestos. This includes tracheal, bronchus, and lung cancer, mesothelioma, pneumoconiosis, larynx, and ovarian cancer, these are all level 3 diseases brought on by occupational exposure to asbestos in the GBD database. The specific ICD codes corresponding to these diseases have been detailed in previous publications [8]. Furthermore, demographic information such as sex and age was collected to estimate the burden of all causes of occupational exposure to asbestos in the U.S.

### Estimation methods

Occupational exposure to asbestos is characterized as the cumulative percentage of individuals subjected to lifelong exposure within an occupational setting. Primary data is sourced from the International Labour Organization, while supplementary datasets are obtained by submitting inquiries to various institutions via the GBD Collaborator Network [18]. Upon data acquisition, consolidation into a tabular format is executed, followed by the generation of survey-weighted estimations of economic activity and occupation across GBD Geography and corresponding years. The Asbestos Impact Ratio (AIR) is then employed to gauge the prevalence of asbestos exposure, calculated as the mortality rate attributed to mesothelioma observed within the population, divided by the mesothelioma mortality rate in individuals highly exposed to asbestos. The AIR is expressed using the following equation:

Mort, Mortality rate due to mesothelioma; N, Mortality rate due to mesothelioma in population not exposed to asbestos; Mort^*^, Mortality rate due to mesothelioma in population highly exposed to asbestos; y, year; c, country; s, sex.

### Statistical analysis

The connection point regression model is utilized to assess the trajectory of disease burden linked to occupational exposure to asbestos in the U.S. from 1990 to 2019. This model is capable of segmenting the entire timeframe and identifying the years (connection points) where the trend experiences significant shifts [19, 20]. Regression analysis is conducted on the natural logarithms of various interval rates, enabling the calculation of the annual percentage change (APC) and its corresponding 95% confidence interval (CI) for each segment. The overall trend is summarized by the annual average percentage change (AAPC) [21–23]. Statistical significance is denoted by a P-value less than 0.05.

We conducted an analysis to highlight the geographic disparities in the number of deaths attributed to occupational exposure to asbestos across various states of the U.S. in 2019. Utilizing relevant maps, we illustrated these differences. Additionally, we examined the distribution of disease burden resulting from occupational exposure to asbestos among different age groups in the same year. Furthermore, we presented the proportion of deaths attributed to occupational exposure to asbestos in 2019, encompassing various conditions such as trace, bruchus, and lung cancer, mesothelioma, pneumoconiosis, larynx cancer, and ovarian cancer. We used Joinpoint Regression Software 4.9, created by the Statistical Research and Applications Branch of the National Cancer Institute in the U.S., to do joinpoint analysis on the data. Subsequently, we utilized R statistical software (version 4.3.2) for further data analysis and visualization.

## Results

### Overall trends of occupational exposure to asbestos

**Fig. 1:**
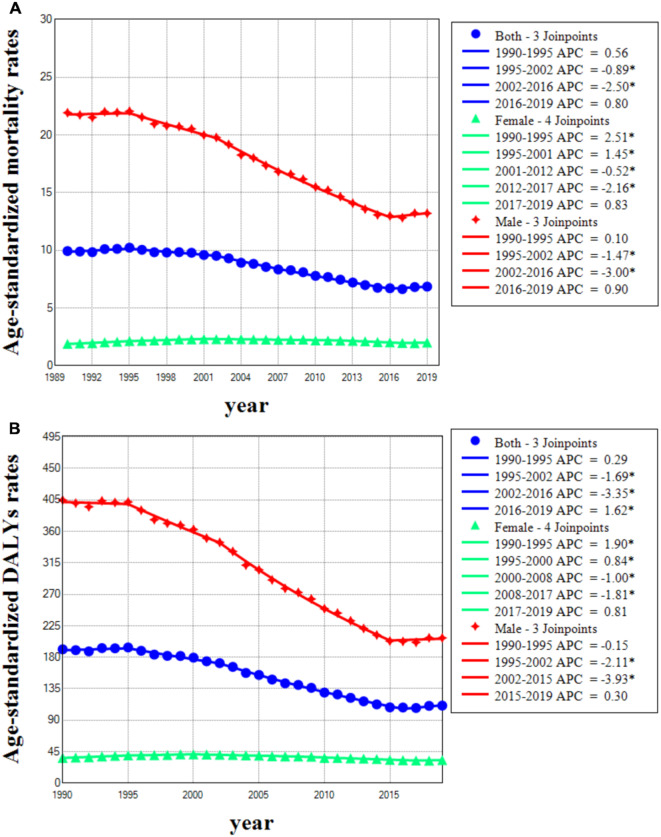
Joinpoint regression analysis in age-standardized mortality (**A**) and DALYs (**B**) rates of all causes occupational exposure to asbestos in the United States from 1990 to 2019 by sexes. DALYs = disability-adjusted life years

In both sexes, the number of deaths attributable to occupational exposure to asbestos increased from 33,926.7 [95% uncertainty interval (UI): 24,871.5–42,661.4] in 1990 to 40,764.1 (95% UI: 30,522.8–51,859.3) in 2019, marking a 20.2% increase. However, ASMR [AAPC − 1.3 (95% CI: -1.5, -1)] and ASDR [AAPC − 1.8 (95% CI: -2, -1.6)] showed varying degrees of decline from 1990 to 2019. Overall, ASMR and ASDR due to occupational exposure to asbestos exhibited a declining trend in males and both sexes, with males showing a more pronounced decrease. Additionally, male ASMR and ASDR were significantly higher than those of both sexes and females. The ASMR of males decreased from 21.94 (95% UI: 15.63–28.17) in 1990 to 13.21 (95% UI: 9.39–17.14) in 2019, marking a -39.8% decrease, with AAPC of -1.7 (95% CI: -1.9, -1.5). Conversely, the ASMR of females increased from 1.9 (95% UI: 1.28–2.57) in 1990 to 2.02 (95% UI: 1.29–2.9) in 2019, representing a 6.3% increase, with AAPC of 0.2 (95% CI: 0, 0.5). Both male and female ASDR showed a decline from 1990 to 2019, with AAPC of -2.3 (95% CI: -2.5, -2.1) and − 0.3 (95% CI: -0.5, -0.1) respectively. Looking at specific time periods, ASMR showed an upward trend from 1990 to 1995 for males, females, and both sexes, followed by a gradual decrease until males and both sexes showed a resurgence in 2016, while females showed a similar resurgence in 2017. ASDR exhibited a similar trend, both sexes shown an increase trend between 1990 and 1995, which was followed by a decreasing trend until both sexes reached 2016, the female started to exhibit an upward trend once more in 2017, while the male showed a negative trend between 1990 and 1995 until 2016 (Fig. 1; Tables 1 and 2).

**Table 1:** Death number, age-standardized mortality rates, and age-standardized DALYs rates of occupational exposure to asbestos in USA in 1990 and 2019

cause	sex	Deaths number			Age-standardized mortality rates per 100,000 population			Age-standardized DALYs rates per 100,000 population		
		1990 No. (95% UI)	2019 No. (95% UI)	Percentage change	1990 No. (95% UI)	2019 No. (95% UI)	Percentage change	1990 No. (95% UI)	2019 No. (95% UI)	Percentage change
All causes	Both	33,926.7 (24,871.5–42,661.4)	40,764.1 (30,522.8–51,859.3)	20.2%	9.94 (7.26–12.54)	6.86 (5.15–8.72)	-31%	191.43 (138.41-245.33)	110.99 (82.46-143.44)	-42%
	Female	3,866.3 (2,597.2-5,259.3)	6,771.2 (4,318-9,705.3)	75.1%	1.9 (1.28–2.57)	2.02 (1.29–2.9)	6.3%	36.3 (24.5-49.59)	33.1 (21.55–47.78)	-8.8%
	Male	30,060.4 (21,389.5–38,821.5)	33,993 (24,120.5–44,187.3)	13.10%	21.94 (15.63–28.17)	13.21 (9.39–17.14)	-39.8%	404.74 (284.49-530.15)	207.7 (145.47-276.13)	-48.7%
Neoplasms	Both	33,609.8 (24,558.6–42,347.8)	40,073.6 (29,867 − 51,090.9)	19.2%	9.85 (7.17–12.45)	6.74 (5.04–8.6)	-31.6%	189.23 (136.29-243.13)	108.75 (80.23-141.22)	-42.5%
	Female	3,851.1 (2,580.9-5,243.6)	6,735.4 (4,297.4-9,656.4)	74.9%	1.89 (1.27–2.56)	2 (1.29–2.89)	5.8%	35.92 (24.07–49.19)	32.81 (21.34–47.45)	-8.7%
	Male	29,758.6 (21,064.4–38,524.4)	33,338.1 (23,427.8–43,535.8)	12%	21.71 (15.39–27.95)	12.95 (9.14–16.88)	-40.4%	399.92 (278.37-525.61)	202.93 (140.46-271.46)	-49.3%
Ovarian cancer	Female	655.9 (285.6-1,052)	931.8 (415-1,545)	42.1%	0.32 (0.14–0.51)	0.28 (0.12–0.45)	-12.5%	5.82 (2.5–9.34)	4.5 (1.93–7.61)	-22.7%
Tracheal, bronchus, and lung cancer	Both	30,463.8 (21,523.4–39,006.8)	35,642.8 (25,661.7–46,332.5)	17%	8.91 (6.27–11.47)	5.99 (4.31–7.78)	-32.8%	170.44 (117.79-224.18)	95.46 (67.06-126.82)	-44%
	Female	2,757.7 (1,667.9-3,973.3)	5,152.8 (3,015.7-7,603.5)	86.9%	1.35 (0.82–1.94)	1.53 (0.9–2.26)	13.3%	25.35 (15.67–36.6)	24.24 (14.47–36.12)	-4.4%
	Male	27,706.1 (19,095.8–36,370.9)	30,490 (20,717.7–40,712.9)	10%	20.19 (13.9-26.45)	11.85 (8.07–15.76)	-41.3%	370.11 (249.05-493.48)	183.87 (121.98-251.84)	-50.3%
Larynx cancer	Both	371.4 (209.1-540.5)	407.2 (222.8-604.6)	9.6%	0.11 (0.06–0.16)	0.07 (0.04–0.1)	-36.4%	2.16 (1.19–3.2)	1.17 (0.65–1.75)	-45.8%
	Female	18.3 (8.6–29.3)	21.9 (10.2–36.3)	19.7%	0.01 (0-0.01)	0.01 (0-0.01)	0	0.18 (0.08–0.29)	0.12 (0.05–0.2)	-33.3%
	Male	353.1 (191-522.2)	385.3 (204.3-582.7)	9.1%	0.26 (0.14–0.38)	0.15 (0.08–0.23)	-42.3%	4.92 (2.63–7.29)	2.48 (1.32–3.81)	-49.6%
Mesothelioma	Both	2,118.7 (1,975.8-2,281.8)	3,091.8 (2,837.9-3,320.7)	45.9%	0.64 (0.59–0.69)	0.53 (0.49–0.57)	-17.2%	13.25 (12.45–14.26)	9.65 (8.86–10.45)	-27.2%
	Female	419.3 (309.6-562.5)	629 (469.8-817.1)	50%	0.22 (0.16–0.29)	0.2 (0.15–0.26)	-9.1%	4.57 (3.21–6.08)	3.96 (2.91–5.51)	-13.3%
	Male	1,699.5 (1,594.4-1,801.7)	2,462.8 (2,278.2-2,611.6)	44.9%	1.26 (1.18–1.33)	0.96 (0.88–1.02)	-23.8%	24.89 (23.53–26.3)	16.59 (15.48–17.58)	-33.3%
Pneumoconiosis	Both	316.9 (287.3-434.4)	690.6 (564.8-753.1)	117.9%	0.09 (0.08–0.13)	0.11 (0.09–0.12)	22.2%	2.21 (1.91–2.77)	2.24 (1.96–2.55)	1.4%
	Female	15.1 (11.4–22.6)	35.7 (19.1–45.1)	136.4%	0.01 (0.01–0.01)	0.01 (0.01–0.01)	0	0.38 (0.25–0.56)	0.29 (0.2–0.37)	-23.7%
	Male	301.8 (271.4-416.1)	654.9 (530.4-717.2)	117%	0.23 (0.2–0.32)	0.26 (0.21–0.28)	13%	4.83 (4.22–6.16)	4.76 (4.15–5.38)	-1.4%

*Note* DALYs, Disability-adjusted life years; UI, uncertainty interval; No., number; USA, United state of Amercia

**Table 2:** The trends of ASMR and ASDR of all causes occupational exposure to asbestos in United states of America by joinpoint regression. ASMR = age-standardized mortality rate; ASDR = age-standardized disability-adjusted life years rate; APC = Annual percent change; AAPC = Average annual percent change

	ASMR				ASDR		
Cohort	Index	Year	Estimate (95% UI)	P value	Year	Estimate (95% UI)	*P-value* value
Both	APC	1990–1995	0.6 (-0.1, 1.2)	0.087	1990–1995	0.3 (-0.3, 0.9)	0.335
		1995–2002	-0.9 (-1.4, -0.4)	0.001	1995–2002	-1.7 (-2.1, -1.2)	0
		2002–2016	-2.5 (-2.7, -2.4)	< 0.001	2002–2016	-3.4 (-3.5, -3.2)	0
		2016–2019	0.8 (-0.6, 2.3)	0.264	2016–2019	1.6 (0.2, 3)	0.024
	AAPC	1990–2019	-1.3 (-1.5, -1)	< 0.001	1990–2019	-1.8 (-2, -1.6)	0
Female	APC	1990–1995	2.5 (2, 3.1)	< 0.001	1990–1995	1.9 (1.4, 2.4)	0
		1995–2001	1.5 (0.9, 2)	< 0.001	1995–2000	0.8 (0.1, 1.6)	0.025
		2001–2012	-0.5 (-0.7, -0.3)	< 0.001	2000–2008	-1 (-1.3, -0.7)	0
		2012–2017	-2.2 (-2.9, -1.4)	< 0.001	2008–2017	-1.8 (-2, -1.6)	0
		2017–2019	0.8 (-1.5, 3.3)	0.474	2017–2019	0.8 (-1.4, 3.1)	0.46
	AAPC	1990–2019	0.2 (0, 0.5)	0.094	1990–2019	-0.3 (-0.5, -0.1)	0.006
Male	APC	1990–1995	0.1 (-0.6, 0.8)	0.768	1990–1995	-0.2 (-0.8, 0.5)	0.631
		1995–2002	-1.5 (-2, -1)	< 0.001	1995–2002	-2.1 (-2.6, -1.6)	0
		2002–2016	-3 (-3.2, -2.9)	< 0.001	2002–2015	-3.9 (-4.1, -3.8)	0
		2016–2019	0.9 (-0.6, 2.4)	0.231	2015–2019	0.3 (-0.6, 1.2)	0.505
	AAPC	1990–2019	-1.7 (-1.9, -1.5)	< 0.001	1990–2019	-2.3 (-2.5, -2.1)	0

### The burden of occupational exposure to asbestos in various states of the United States in 2019

**Fig. 2:**
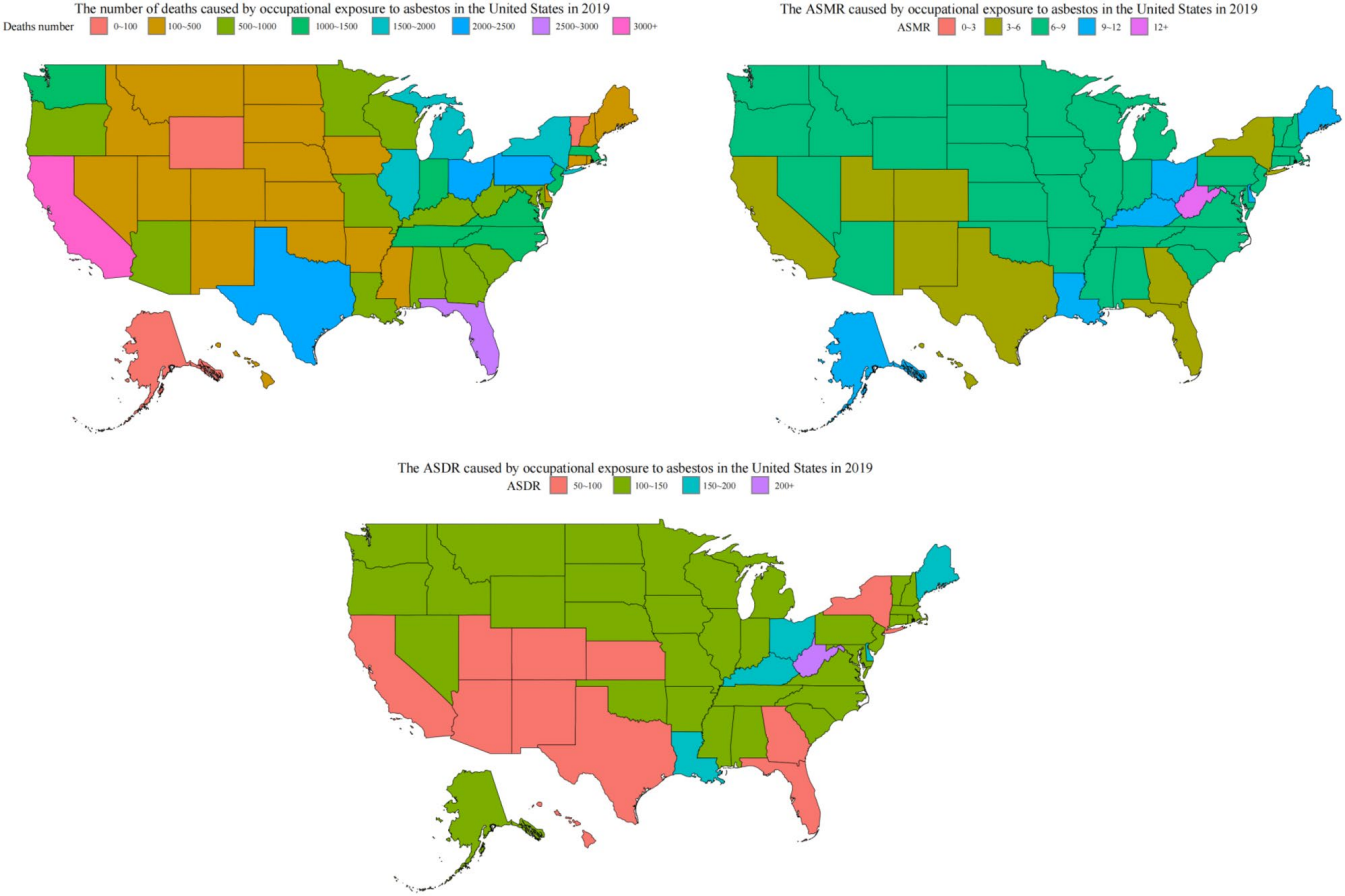
The number of deaths, ASMR, and ASDR caused by occupational exposure to asbestos in the United States in 2019

Figure 2 illustrates the fatalities, ASMR, and ASDR caused by occupational exposure to asbestos in each state during 2019. California reported the highest number of deaths, with 3,151 fatalities, followed closely by Florida with 2,775.9, Pennsylvania with 2,357.7, Texas with 2,343.5, and Ohio with 2,061.1. Conversely, the District of Columbia reported the lowest number of fatalities, totaling 28.5 (Fig. 2 and Additional file 2). In 2019, West Virginia, Maine, and Louisiana recorded the highest age-standardized mortality rates (ASMR) at 12.4, 11.3, and 10.5 respectively, compared to the lowest rate in the District of Columbia at 3 per 100,000 population (Fig. 2 and Additional file 3). Similarly, the highest age-standardized disability-adjusted life years (ASDR) rates were observed in West Virginia at 218.3, Maine at 186.5, and Louisiana at 182. Contrastingly, the District of Columbia again reported the lowest rate at 50.9 per 100,000 population (Fig. 2 and Additional file 4).

### Age and sex patterns

**Fig. 3:**
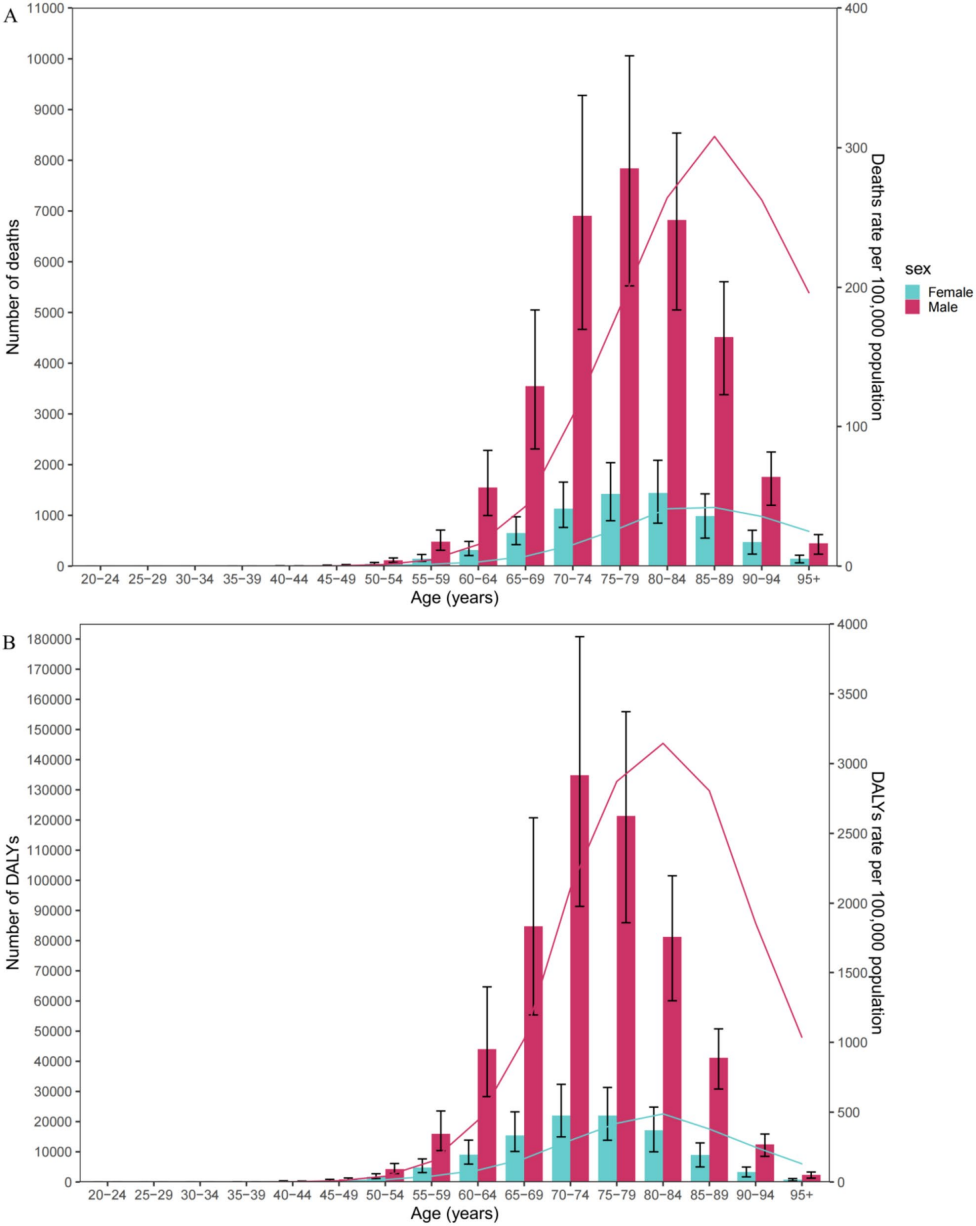
Number of deaths and death rate (**A**) and number of DALYs and DALYs rate per 100,000 population (**B**) of all causes occupational exposure to asbestos by age and sex in 2019. The bar charts are numbers and the lines are rate. DALY, disability-adjusted life years

From 1990 to 2019, the number of deaths and DALYs was lower in females compared to males. The number of death cases gradually increased starting from the age group of 50–54 years and continued to rise with age, reaching its peak in males aged 75–79 years, after which it began to decline rapidly. A similar pattern was observed in females, with the peak in the number of deaths occurring in the 80-84-year-old age group. Additionally, the number of DALYs increased with age, peaking in males aged 70–74 years, followed by a substantial decrease. In females, the number of age-specific DALYs peaked in the 75-79-year-old age group. Age-specific death rates increased from the age group of 50–54 years to 85–89 years in both genders, after which they rapidly declined in males. Similarly, age-specific rates of DALYs increased from the age group of 50–54 years to 80–84 years in both genders, followed by a rapid decline in males (Fig. 3).

### Different causes of death

**Fig. 4:**
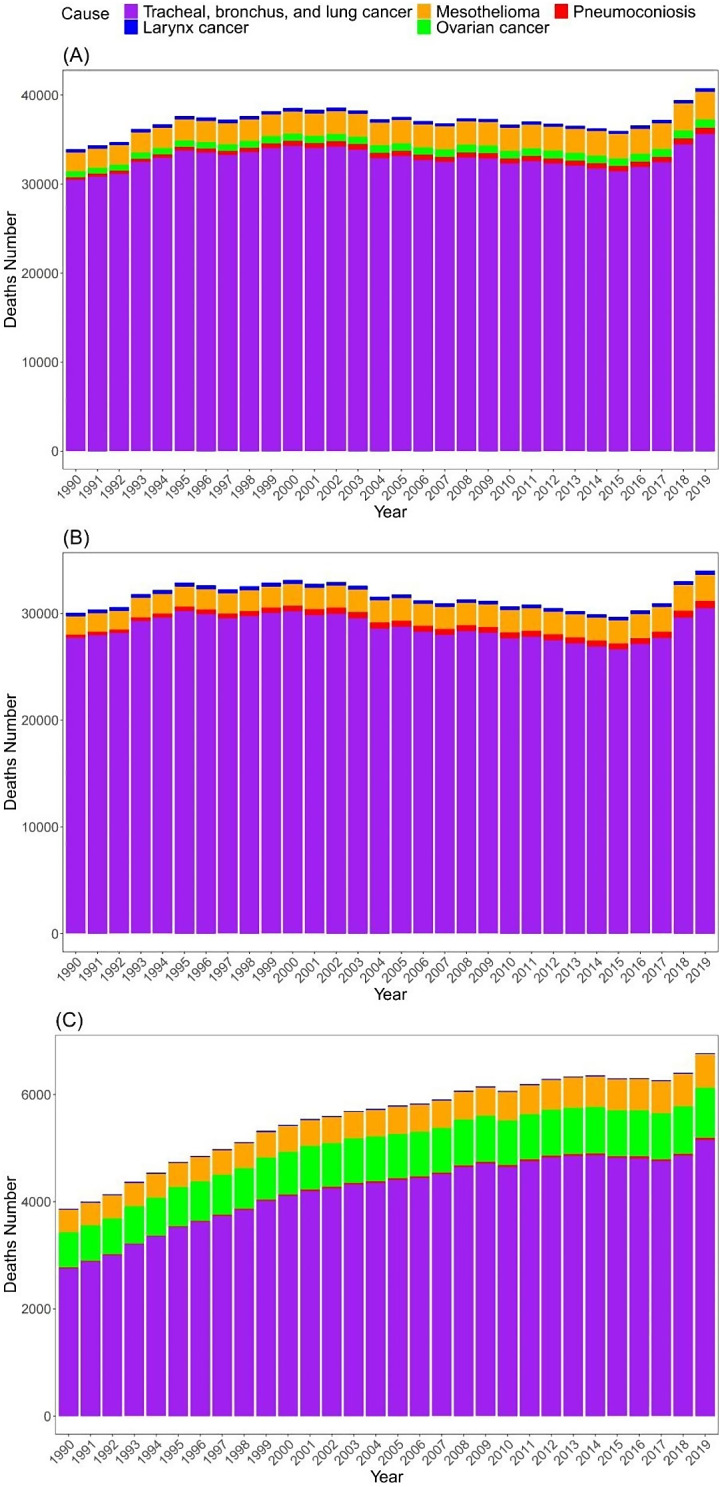
Deaths number of tracheal, bronchus, and lung cancer, mesothelioma, pneumoconiosis, larynx cancer, and ovarian cancer occupational exposure to asbestos in both sexes (**A**), males (**B**), and females (**C**) in United states of America from 1990 to 2019

Overall, tracheal, bronchus, and lung cancer account for the highest number of deaths attributed to occupational exposure to asbestos, and it appears that these numbers have been steadily increasing over the past five years. In both sexes and males specifically, mesothelioma ranks as the second leading cause of death, while ovarian cancer ranks second among females. Notably, pneumoconiosis significantly contributes to male deaths but less so to female deaths (Fig. 4).

In the U.S., among both sexes, neoplasms account for the highest number of deaths attributable to occupational exposure to asbestos. The number has increased from 33,609.8 (95% UI: 24,558.6–42,347.8) in 1990 to 40,073.6 (95% UI: 29,867–51,090.9) in 2019, representing a 19.2% increase. Pneumoconiosis, causing deaths, has seen a substantial rise from 316.9 (95% UI: 287.3–434.4) in 1990 to 690.6 (95% UI: 564.8–753.1) in 2019, marking a 117.9% increase. Within neoplasms, deaths attributed to tracheal, bronchus, and lung cancer have increased from 30,463.8 (95% UI: 21,523.4–39,006.8) in 1990 to 35,642.8 (95% UI: 25,661.7–46,332.5) in 2019, marking a 17% increase. From 1990 to 2019, death counts due to different causes have increased to varying extents. Notably, pneumoconiosis-related deaths in female patients have seen the highest percentage increase at 136.4%, whereas deaths in male patients due to larynx cancer have seen the lowest percentage increase at 9.1%.

Regarding ASMR, the percentage change in deaths attributed to neoplasms (5.8%) and tracheal, bronchus, and lung cancer in females has increased, as well as pneumoconiosis in both sexes (22.2%) and males (13%). However, the rest have decreased, with the largest decline observed in the percentage change of larynx cancer in males at -42.3%, and the smallest decline seen in the percentage change of mesothelioma in females at -9.1%. In ASDR, apart from a 1.4% increase in the percentage change attributed to pneumoconiosis in both sexes, the rest have decreased. The largest decline is observed in males with tracheal, bronchus, and lung cancer at -50.3%, followed by larynx cancer in males at -49.6% (Table 1).

## Discussion

In this study, we report the estimated disease burden attributable to occupational exposure to asbestos in the U.S. as estimated by GBD 2019. According to our research, there was a rise in asbestos-related workplace mortality in the United States between 1990 and 2019, despite a decline in ASMR and ASDR over the same period. In age groups greater than 50–54, males have higher mortality numbers, ASMR, and ASDR than females, and the burden reaches its peak among the elderly. Furthermore, the primary causes of mortality from asbestos exposure at work are lung cancer, trachea, and bronchus, and the number of deaths from these conditions has been increasing recently. However, it is imperative to acknowledge the persistent burden of asbestos-related diseases, highlighting the ongoing need for preventive measures and targeted interventions in the U.S.

Exposure to asbestos may lead to lung cancer, laryngeal cancer, ovarian cancer, and mesothelioma. Additionally, exposure to asbestos can also lead to other diseases such as asbestosis. An incurable disease of the serosal lining, malignant mesothelioma is frequently brought on by asbestos exposure [24]. Pneumoconiosis includes silicosis, asbestosis, coal worker`s pneumoconiosis, and other pneumoconiosis [25]. We know that a significant portion of the global cancer burden may be prevented through interventions aimed at reducing exposure to known cancer risk factors [26]. As early as 2007, the World Health Organization (WHO) formulated the national program outline for eliminating asbestos-related diseases [27]. According to the American Mineral Commodity Summaries 2024, the estimated global consumption of unmanufactured asbestos fibers from 2015 to 2023 was 1.1 to 1.3 million metric tons per year [17]. However, it is estimated that every 20 metric tons of asbestos produced and consumed globally leads to one death [6]. We must recognize that the most effective way to eliminate diseases related to asbestos is to stop using all types of asbestos.

In this study, we focused on assessing the trends and burden of occupational exposure to asbestos in the U.S. from 1990 to 2019. We found that both ASMR and ASDR decreased to varying degrees in both males and females as well as in both sex groups. This discrepancy may be attributed to several factors. Firstly, advancements in medical treatment and diagnostic techniques may have contributed to improved survival rates among individuals diagnosed with asbestos-related diseases, thus influencing the observed decline in ASMR and ASDR [28]. Additionally, changes in occupational safety regulations and increased awareness of asbestos-related hazards may have led to reduced exposure levels over time, although the lag effect of historical exposure cannot be overlooked. Around 40 years ago, a research was carried out by the National Research Council (NRC) in Atlanta, Georgia, to determine the relative risk of negative health consequences from exposure to several environmental mutagenic pollutants (EMPs). After examining several EMP kinds, it was determined that exposure to chrysotile asbestos posed the most danger, primarily due to the greater likelihood of airborne particles of respirable size being inhaled. This finding highlights the importance of addressing and mitigating the risks associated with chrysotile asbestos exposure in order to protect public health [29, 30]. Gutiérrez et al. provide guidelines for the removal and ultimate disposal of installed asbestos-containing construction materials. These recommendations can be helpful in directing national and regional organizations as they create comprehensive plans with precise, quantifiable, and doable objectives for the replacement of installed asbestos-containing building materials in the future [31].

Our comprehensive regression analysis reveals that from 1990 to 2019, both ASMR and ASDR of all causes occupational exposure to asbestos have declined across all genders. However, a notable increase of APC has been observed since 2016 for both sexes, and particularly among females, starting in 2017. Despite only the ASDR indicating statistical significance with a P-value of less than 0.05, this trend warrants our attention. This may be attributed to a consistent rise in pneumoconiosis cases [32]. Interestingly, an investigation into the incidence and prevalence rates of pneumoconiosis among U.S. medical insurance beneficiaries from 1999 to 2019 found a decline in asbestosis cases, yet pneumoconiosis remains prevalent among medical insurance beneficiaries [33]. However, the GBD database did not take into account the impact of pneumoconiosis on asbestosis data, which may lead to overestimation of the burden on asbestosis.

Our analysis revealed significant variations in the number of deaths, ASMR, and ASDR caused by occupational exposure to asbestos across different states in the U.S. in 2019. California reported the highest number of fatalities, followed by Florida, Pennsylvania, Texas, and Ohio. The three states with the highest ASMR and ASDR are West Virginia, Maine, and Louisiana. These disparities may reflect differences in industrial activities, historical asbestos use, and occupational safety regulations among states. For instance, states with a legacy of heavy industry and manufacturing may experience higher rates of asbestos-related diseases due to prolonged exposure among workers. The chlor-alkali industry is the only remaining major consumer of asbestos minerals in the U.S. The good news is that in 2023, three out of eight chlor-alkali plants in the U.S. are using alternative materials to replace asbestos diaphragms [6]. Many materials can replace asbestos, including calcium silicate, carbon fiber, etc. Some non-fibrous minerals or rocks, such as perlite, are also considered possible alternatives to asbestos.

Our research findings emphasize different age and gender patterns in the burden of asbestos-related diseases in the U.S. Overall, the disease burden caused by occupational exposure to asbestos in males is much higher than in females. This may be related to a greater chance of asbestos exposure for males. This obliquely implies that females are less likely than males to be exposed to asbestos. The age group with the highest number of male deaths is 75–79 years old, which is related to the long incubation period of diseases caused by asbestos exposure. The mortality rate for males and females reaches its peak in the 85–89 age group, after which the mortality rate for males rapidly decreases while the rate for females steadily decreases. This may reflect differences in susceptibility to asbestos-related diseases and healthcare-seeking behaviors between males and females. Therefore, screening the asbestos exposure history of elderly cancer patients, establishing a specialized registry, and early diagnosis and treatment of asbestos-related diseases are also crucial [4].

Tracheal, bronchus, and lung cancer emerged as the leading causes of death attributed to occupational exposure to asbestos, with a notable increase in number of deaths observed over the past five years. The second most common cause of mortality for both sexes was mesothelioma, whereas ovarian cancer was the second most common cause of death among females who had worked with asbestos. The increasing trend in number of deaths for these diseases underscores the persistent health risks associated with asbestos exposure and emphasizes the need for continued surveillance and preventive measures. Furthermore, the differential impact of asbestos-related diseases across genders highlights the importance of tailored approaches to disease management and prevention. Based on the perspective of Markowitz, it is believed that promoting low-dose computed tomography (LDCT) screening among asbestos-exposed workers is of utmost importance. Markowitz also suggests certain standards for LDCT screening. Moreover, the requirements also cover people who have (a) smoked for a minimum of ten pack-years without a time limit following their cessation, or (b) have a history of asbestos-related fibrosis, chronic lung disease, a family history of cancer, a personal history of cancer, or exposure to numerous workplace lung carcinogens [28]. In addition to focusing on tracheal, bronchus, and lung cancers caused by occupational asbestos exposure, we should also pay attention to ovarian cancer caused by occupational asbestos exposure among females. Furthermore, it is important to pay more attention to ASMR, which has demonstrated an increasing tendency in both sex and male, and the fact that the annual number of deaths from pneumoconiosis has more than doubled since 1990.

There are still some limitations to this study. First, GBD data is reconstructed through mathematical models from different quality data sources, which inevitably has some limitations and is difficult to avoid bias. Second, we use the Joinpoint regression model to determine the time trends of the entire period and each segmented period, but there are also some limitations to this model. Third, our study did not assess the potential effects of asbestos exposure in non-occupational asbestos exposure. Fourthly, there are no precise data in the GBD database, it is inferred using the model, it still needs to be confirmed what the real scenario is. Finally, there may be differences in the early identification of diseases, thus there may be biases present.

## Conclusion

In summary, our research offers important new understandings of the patterns and costs associated with asbestos exposure at work in the U.S. Although the disease-related mortality rates caused by occupational asbestos exposure in the U.S. have been decreasing, the number of deaths has been continuously increasing in recent years, especially for tracheal, bronchus, and lung cancers. We advocate for the U.S. to immediately implement a total prohibition on asbestos.

### Electronic supplementary material

Below is the link to the electronic supplementary material.

Supplementary Material 1

Supplementary Material 2

Supplementary Material 3

Supplementary Material 4

## Data Availability

The datasets presented in this study can be found in online database. The names of the database can be found below: ghdx.healthdata.org/gbd-results-tool.
