# Supplementary material for: Assessing trends and burden of occupational exposure to asbestos in the United States: a comprehensive analysis from 1990 to 2019
Source: BMC Public Health. 2024 May 27;24:1404. doi: 10.1186/s12889-024-18919-7 (PMC11129425; doi:10.1186/s12889-024-18919-7)
Supplement: Supplementary file 1 — Supplementary Material 1 [file 12889_2024_18919_MOESM1_ESM.docx]

Trend data parameter settings for the entire United States from 1990 to 2019: "Risk factor" for the GBD estimate; "deaths, DALYs" for measure; "number and rate" for metric, "occupational exposure to asbestos" for risk, "All causes" for cause, "United States of America" for location, "all ages and age-standardized" for age, and "1990–2019" for year.

Data parameter settings for various states in the United States in 2019: "Risk factor" for the GBD estimate; "deaths, DALYs" for measure; "number and rate" for metric, "occupational exposure to asbestos" for risk, "All causes" for cause, "Alabama, Alaska, Arizona, Arkansas, California, Colorado, Connecticut, Delaware, Florida, Georgia, Hawaii, Idaho, Illinois, Indiana, Iowa, Kansas, Kentucky, Louisiana, Maine, Maryland, Massachusetts, Michigan, Minnesota, Mississippi, Missouri, Montana, Nebraska, Nevada, New Hampshire, New Jersey, New Mexico, New York, North Carolina, North Dakota, Ohio, Oklahoma, Oregon, Pennsylvania, Rhode Island, South Carolina, South Dakota, Tennessee, Texas, Utah, Vermont, Virginia, Washington, West Virginia, Wisconsin, Wyoming, District of Columbia." for location, "all ages and age-standardized" for age, and "2019" for year.

Analysis of differences in different age groups in 2019: "Risk factor" for the GBD estimate; "deaths, DALYs" for measure; "number and rate" for metric, "occupational exposure to asbestos" for risk, "All causes" for cause, "United States of America" for location, "20-24, 25-29, 30-34, 35-39, 40-44, 45-49, 50-54, 55-59, 60-64, 65-69, 70-74, 75-79, 80-84, 85-89, 90-94, 95+" for age, "female, and male" for sex, and "1990–2019" for year.

Analysis of Different Gender and Diseases："Risk factor" for the GBD estimate; "deaths" for measure; "number" for metric, "occupational exposure to asbestos" for risk, "Ovarian cancer, Pneumoconiosis, Larynx cancer, Tracheal, bronchus, and lung cancer, Mesothelioma" for cause, "United States of America" for location, "all ages" for age, "both, female, and male" for sex, and "1990–2019" for year.
